# Putting the health in hidden Markov models: incorporating allostatic load indices into movement ecology analyses

**DOI:** 10.1093/conphys/coaf022

**Published:** 2025-04-11

**Authors:** Courtney R Shuert, Marie Auger-Méthé, Karine Béland, Nigel E Hussey, Marion R Desmarchelier, Marianne Marcoux

**Affiliations:** Arctic Aquatic Research Division, Freshwater Institute, Fisheries and Oceans Canada, 501 University Crescent, Winnipeg, Canada, R3T 2N6; Conservation and Research, Assiniboine Park Conservancy, 2595 Roblin Blvd., Winnipeg, Canada R3P 2N7; Department of Statistics, University of British Columbia, 2207 Main Mall, Vancouver, BC, Canada, V6T 1Z4; Institute for the Oceans & Fisheries, University of British Columbia, 2202 Main Mall, Vancouver, BC, Canada, V6T 1Z4; Department of Clinical Sciences, Faculté de Médecine Vétérinaire, Université de Montréal, 3200 rue Sicotte, Montreal, Canada, J2S 2M2; Department of Integrative Biology, University of Windsor, 401 Sunset Ave., Windsor, ON, Canada, N9B 3P4; Department of Clinical Sciences, Faculté de Médecine Vétérinaire, Université de Montréal, 3200 rue Sicotte, Montreal, Canada, J2S 2M2; Arctic Aquatic Research Division, Freshwater Institute, Fisheries and Oceans Canada, 501 University Crescent, Winnipeg, Canada, R3T 2N6

**Keywords:** Allostatic load, animal health, behaviour, hidden Markov models, narwhal, stress

## Abstract

Individual animal health assessments are a key consideration for conservation initiatives. Environmental shifts associated with climate change, such as documented rises in pathogen emergence, predation pressures and human activities, create an increasingly stressful world for many species and have been linked with marked changes in movement behaviour. Even in healthy individuals, variations in allostatic load, the cumulative effects of long-term stress, may alter behavioural priorities over time. Here, we aimed to build links between animal health assessment information and movement ecology, using narwhals in the Canadian Arctic as a case study. A composite stress index was developed to incorporate multiple available health (e.g. health assessments), stress (e.g. hormones) and body condition metrics from clinically healthy individuals, and applied within the framework of widely used hidden Markov modelling of animal movement data. Individuals with a higher composite stress index tended to prioritize behaviours indicative of a stress response, including increasing the probability of transitioning to transiting behaviour as compared to those with a lower stress index. By incorporating a composite stress index that synthesizes multiple health indices in a flexible framework, we highlight that including information indicative of allostatic load may be important in explaining variation in behaviour, even for seemingly healthy animals. The modelling framework presented here highlights a flexible approach to incorporate health assessment information and provides an approach that is widely applicable to existing and future work on a range of species.

## Introduction

It is increasingly recognized that animal health and stress monitoring is crucial to understanding and managing species (Ruscio *et al.*, 2015). Stress and animal health are closely connected with many overlapping and complex mechanisms impacting individuals (Seeley *et al.*, 2022), hindering their incorporation into modelling frameworks. Adaptations that allow animals to adjust to stressors through allostasis, the adaptive link between physiology and the environment (Romero *et al.*, 2009), can benefit individuals in the short term; however, repeated, cumulative stress responses can increase allostatic load (Edes *et al.*, 2018). Field assessments of animal health are commonly gathered for many species (Kophamel *et al.*, 2021), but the logistics and priorities of sampling individuals often vary by species, project and annual goals. Even within a single project, complications with the collection of parameters may also vary due to the ability, e.g. to sample sufficient quantities of blood and other tissue samples or morphometric measurements. Despite the importance of these few data points in certain species, small sample sizes often limit a researcher’s capacity to apply statistical analyses on individual health parameters. Therefore, a flexible framework that can incorporate multiple health parameters and deal with missing data is needed to simplify the complex array of health and stress information collected across a number of individuals. One such method for evaluating multiple, complex dimensions of animal health and stress is by compiling an allostatic load index, an approach commonly used to assess chronic (long-term) stress in humans (e.g. Robertson *et al.*, 2017) and, more recently, in non-human animals (Edes *et al.*, 2021; Seeley *et al.*, 2022).

Climate change is driving rapid changes to both the abiotic and biotic environment, such as increasing the frequency of extreme weather events, decreasing summer sea ice extent (Wassmann *et al.*, 2011) and modifying prey base composition (Fossheim *et al.*, 2015; Ardyna and Arrigo, 2020; Florko *et al.*, 2021) while altering predator–prey relationships (Hamilton *et al.*, 2017; Lefort *et al.*, 2020). Human impacts on landscapes are increasing (Halpern *et al.*, 2015; Halpern *et al.*, 2019). As stressors compound in this environment, reports of novel pathogens (Van Bressem *et al.*, 2009; Buhler *et al.*, 2020; Nielsen *et al.*, 2023), disease outbreaks (VanWormer *et al.*, 2019; Mavrot *et al.*, 2020) and changes in body condition (Galicia *et al.*, 2020; Vermeulen *et al.*, 2023) in many terrestrial and aquatic species continue to surface. Such impacts on animal health are likely to cause fluctuations or declines in population trajectories and modify associated movement patterns (Schick *et al.*, 2013).

Climate-driven changes in movement patterns (Hauser *et al.*, 2021; Szesciorka and Stafford, 2023), migratory phenology (Cherry *et al.*, 2013; Hauser *et al.*, 2017; Pendleton *et al.*, 2022; Shuert *et al.*, 2022; Cooke *et al.*, 2024) and behaviour (as reviewed in Brandt *et al.*, 2023) have been documented through electronic tracking studies. Studies investigating the impact of health and stressors in the environment have focused on evaluating mechanistic linkages between animal treatment groups exposed to acute stressors, such as seismic (Gordon *et al.*, 2003; Heide-Jørgensen *et al.*, 2021) or sonar testing dose experiments (DeRuiter *et al.*, 2013; Harris *et al.*, 2018), or to more chronic stressors, such as entanglements (Pirotta *et al.*, 2023), shipping noise (Rolland *et al.*, 2012) or disease (Holser *et al.*, 2023). However, what might appear to be smaller scale stress responses in comparison (e.g. shifts in body condition or health parameters) have also been found to impact behavioural energetics (Jeanniard du Dot *et al.*, 2009; Pagano *et al.*, 2018; Spitz *et al.*, 2019). Despite the potentially important repercussions of such shifts in behaviours, little work has been undertaken to directly incorporate health assessments into movement models for clinically healthy individuals.

Here, we aimed to develop and extend a flexible framework borrowed from allostatic load indices for incorporating multiple health, stress and body condition metrics into a movement ecology analysis. As a case study, we use data collected from a culturally important Arctic cetacean, the narwhal (*Monodon monoceros*), which is considered a species of concern given the ongoing effects of climate change and anthropogenic stressors in their environment (Tervo *et al.*, 2023). Using satellite telemetry data from narwhal in a hidden Markov model (HMM) framework, we also aimed to advance our understanding of links between composite stress indices and movement behaviour.

## Materials and Methods

### Satellite telemetry devices

Narwhal were captured in Tremblay Sound (72^o^21.389 N, −81^o^05.855 W) at the northern end of Baffin Island, Nunavut, Canada, in 2017 and 2018. The tagging effort, led by Fisheries and Oceans Canada’s long-term marine mammal monitoring program, was undertaken in order to monitor the health and movement of narwhal. Narwhal were handled according to standardized protocols outlined elsewhere (Orr *et al.*, 2001; Shuert *et al.*, 2021). Each narwhal was outfitted with a TDR10 satellite transmitting tag (Wildlife Computers, Inc.), which provided Fastloc GPS-enabled location data (*n* = 10). Satellite telemetry devices were also equipped with time-depth recorders which sampled depth (in metres) every 75 s. Each satellite transmitter was mounted onto the dorsal ridge of a narwhal using a three-pin and spider wire configuration for long-term attachment (see Shuert *et al.*, 2021). This study took place largely within the month of August, where the mean daylight hours are 19.5 h (range 24–16 h at the start and end of the month). All capture and tagging protocols were approved by the Fisheries and Oceans Canada Animal Care Committee, and a Licence to Fish for Scientific Purposes was granted.

### Location and dive data processing

As our goal was to understand the influence that allostatic load may have on narwhal during the summering period, we only considered location and dive data within 30 days of capture/release. The first 24-h post-handling of all data was excluded from analysis to account for any short-term capture-related changes in movement patterns, as has been demonstrated elsewhere for these tagged individuals (Shuert *et al.*, 2021). While satellite telemetry devices transmitted Argos location services, here we only consider the more accurate Fastloc GPS locations. Location data for each individual’s track was estimated and corrected for error by fitting a continuous-time correlated random walk within the package ‘crawl’ in R (Johnson *et al.*, 2008; Johnson and London, 2022). This procedure estimated locations at 1-h intervals (n = 6677 locations), chosen to balance the frequency of location information while minimizing the presence of gaps between subsequent intervals. The distance and angle between consecutive locations was used to derive a data stream of step lengths and turning angles, respectively. Where gaps in raw location data exceeded 3 h, estimated step lengths and turning angles between model-derived locations for these periods were removed to prevent bias in estimating behavioural states from a movement path derived from ‘crawl’ when interpolating additional points between known locations (*n* = 4985 locations remaining).

We used the ‘diveMove’ package in R (Luque, 2007) to process the raw dive data and extract two behaviourally relevant dive metrics. First, we applied a zero-offset correction to handle any drift in depth sensor measurements following (Luque and Fried, 2011) and retained only dives ≤10 m depth as we considered diving behaviour above this threshold to be behaviour at the surface. The ‘diveMove’ package calculates several metrics for each individual dive, but we focused on maximum dive depth (in metres) and dive wiggles (in metres), the latter of which is defined as the sum of all depth changes during the bottom phase of each dive (Luque, 2007). Narwhal are highly specialized deep divers (Laidre *et al.*, 2003), consequently, these two metrics indicate diving ability (maximum depth) and foraging effort at depth (i.e. more dive wiggles are assumed to equate to increased foraging effort at depth), respectively.

Distance to shore has previously been shown to be an important factor determining a narwhal’s refuging location and behavioural response in a stressful scenario (Breed *et al.*, 2017; Heide-Jørgensen *et al.*, 2021), and was therefore included as an additional data stream in our HMM. For each estimated location, distance to shore (in kilometres) was extracted from MARSPEC at a gridded resolution of 0.083 degrees by bilinear interpolation between grid cells (Sbrocco and Barber, 2013).

### Narwhal health data collection

All narwhals were examined by veterinary staff during capture-tagging and blood samples as well as a broad range of information related to health and condition metrics to compile a composite health index. We recorded additional health-related information where possible, including such information as the sex of the individual and when females interacted with calves during and after handling events. Interactions with calves were considered an important variable, as narwhal may nurse young beyond 2 years (Zhao *et al.*, 2021). As such, their protracted lactation burden and the potential additive stress response of being separated from their calves during the capture-tagging process likely contribute to an individual’s stress load. We also included the presence of ectoparasites (likely *Cyamiid* lice) as a potential health parameter (Schick *et al.*, 2013), accepting that their detrimental impact is likely low (Hermosilla *et al.*, 2015). We acknowledge that all individuals likely have some level of parasite load not visible during examination, but we assume here that individuals with visible ectoparasites likely had higher levels than others. Predator sightings (namely killer whale, *Orcinus orca*; see Breed *et al.*, 2017) at the time of capture were also noted. For each individual, standard length (in centimetres) was measured from the tip of the rostrum to the notch in the tail fluke posterior to the peduncle. Axillary girth was also taken where possible as the circumference of the body immediately posterior to the pectoral flippers (in centimetres). Age class was determined by standard length, where juveniles and adult whales were split by being below or above a standard length of 300 cm, respectively (Hay, 1984). The above data were used to establish a body condition index following Choy *et al.* (2019) in which the residuals are used from the best fitting model predicting half-girth with standard length, age class and sex as predictors. Narwhals with positive residuals were considered to have higher-than-average body condition, while those with negative residuals were considered to have lower-than-average body condition (Choy *et al.*, 2019).

Blood samples were attempted for each narwhal (Béland *et al.*, 2023). In brief, blood samples were collected from the dorsal or ventral fluke veins with a 19-gauge butterfly and syringe. Each sample was transferred to a 3-ml heparinized tube, then transferred to a 10-ml serum separator tube and centrifuged for 10 min at 1000 *g*. Plasma and serum samples were then aliquoted into cryovials and stored at −20°C in the field in a freezer for up to 3 weeks before being transferred to long-term storage at −80°C. As outlined in Béland *et al.* (2023), serum samples were used to measure stress hormones, including dehydroepiandrosterone (DHEA) and its sulphated metabolite (DHEA-S) and cortisol. DHEA and DHEA-S were measured using enzyme-linked immunosorbent assays (ELISA; CAN-DHS-490 and CAN-DHS-480, Diagnostic Biochem Canada Inc., London, ON, Canada), while cortisol was measured using a solid-phase competitive chemiluminescence enzyme immune-assay (IMMULITE^®^ 1000, Siemens Healthcare Limited, Oakville, ON, Canada). If two blood samples were taken at both the start and end of the handling event, we considered the mean hormone value of these two estimates. Cortisol is a catabolic glucocorticoid hormone and a commonly measured biomarker of stress in marine mammals, including narwhal (Watt *et al.*, 2021), and DHEA and DHEA-S are anabolic androgen hormone precursors with protective and regenerative roles. Here, we considered cortisol to be a measure of acute stress, while the ratio of cortisol to DHEA as well as DHEA-S provides a potential indicator of chronic stress, as has been documented in other species (Krause *et al.*, 2014; Gundlach *et al.*, 2018; Fusi *et al.*, 2021; Miller *et al.*, 2021). We considered individuals to be stressed if measured levels of cortisol, DHEA-S and the ratio of cortisol to DHEA was found to be >42.3 ng ml^−1^, <5.75 ng ml^−1^ and a ratio >98.2, respectively. These thresholds were based on values being in the upper or lower quartiles across all measurements for all individuals. The upper quantile was chosen for cortisol and cortisol:DHEA ratio as these tend to be elevated in acute stressful scenarios (Béland *et al.*, 2023; Wolfe and Edes, 2023), while the lower quartile was selected for DHEA-S as it tends to be downregulated by chronic stress (Beer *et al.*, 2023). The quartile approach to quantifying allostatic load index does not necessarily equate to clinical thresholds, but instead aims to capture pre-clinical assessments of relative risk for increasing stress (Edes *et al.*, 2018).

### Composite stress index

To test a flexible framework for incorporating the multiple dimensions of health and stress for modelling purposes, a composite stress index was developed to highlight relative, compounding stressors. Following the allostatic load index approach (as reviewed in Seeley *et al.*, 2022), a simple scaled additive approach was adopted to allow for flexibility of incorporating known information (when measured) on health and stress, while taking into account relative severity of the various parameters. Stress index parameters for narwhal were scored as follows: (i) the presence of healed wounds characteristic of injuries related to previous trauma (yes = 0.5, no = 0), (ii) the presence of fresh wounds that were unrelated to capture or handling and likely as a result of recent trauma (yes = 2, no = 0), (iii) the presence of ectoparasites (e.g. cyamid lice, likely *Cyamus* spp., yes = 1, no = 0), (iv) the presence of predators in the area at time of capture (yes = 1 only when present), (v) the presence of an accompanying calf in females (yes = 1, no = 0 for females), (vi) negative body condition index residual values (yes = 1, no = 0), (vii) measured cortisol values >42.3 ng ml^−1^ (yes = 1, no = 0), (viii) measured DHEA-S values <5.75 ng ml^−1^ (yes = 1, no = 0) and (ix) the measured ratio of cortisol to DHEA >98.2 (yes = 1, no = 0).

All parameters were weighted equally following convention with allostatic load indices (e.g. Seeley *et al.*, 2021), with the exception of the presence of healed scars (weighted as half) and the presence of active wounds unrelated to capture (weighted as double). The sum of each parameter was calculated following the general approach to developing an allostatic load index. However, to account for the availability of each parameter in a given narwhal (i.e. some individuals were missing data), the total score was scaled against the available dimensions (the sum of parameter scores was divided by the number of parameters for which data were available) to give a relative index of an individual stress load, representing a composite of possible additive stressors. Individuals with composite stress index scores closer to 1 were considered to be more stressed than individuals with values closer to 0. A summary of this composite stress index is highlighted in Table 1 for individuals included in this study. An alternative formulation of the composite stress index using only healed scars, active wounds and the presence of ectoparasites where data were available for all individuals included in the modelling framework (i.e. no missing data) is explored in the Supplementary Materials.

**Table 1:** Composite stress index

ID	Length	Sex	(a) Healed scars	(b) Wound	(c) *Cyamids*	(d) KW	(e) Calf	(f) BCI	(g) Cort.	(h) DHEA-S	(i) Cort: DHEA	Total Score	# Dims	**CSI**
MM-01	466	M	0	0	0				0	0	0	0	6	**0.00**
MM-02	400	F	0	0	0		0					0	4	**0.00**
MM-03	400	F	0.5	0	0		0	1	0	0	0	1.5	8	**0.19**
MM-04	432	M	0.5	2	0			0	1	0	1	4.5	7	**0.64**
MM-05	488	M	0.5	0	1				1	0		2.5	5	**0.50**
MM-06	458	M	0.5	2	0				0	1	0	3.5	6	**0.58**
MM-07	430	M	0.5	0	0			0				0.5	4	**0.13**
MM-08	375	F	0	0	0		0	0				0	5	**0.00**
MM-12	425	F	0.5	0	1	1	1	1	1	0	1	6.5	9	**0.72**
MM-13	298	M	0	0	0							0	3	**0.00**

Health and stress metrics gathered from narwhal during capture-tagging and used to calculate the composite stress index (CSI, highlighted in bold). Variables, including the presence of (a) healed scars, (b) active wounds, (c) ectoparasites, (d) predators (KW), (e) calves, (f) negative body condition index (BCI) residuals and elevated levels of hormones including (g) cortisol (h) DHEA-S and (i) cortisol/DHEA ratio. Each stress index parameter was scored by relative severity, when measured. The total additive score across parameters was then scaled according to the number of available dimensions (# dims).

### Statistical analysis

To understand how differing relative stress load may impact movement, we used an HMM to classify movement behaviour and link movement behaviours with our novel composite stress index. An HMM is a hierarchical model that simultaneously models a time series of observations and an underlying, usually hidden, state sequence (Zucchini and MacDonald, 2009). In movement ecology, these hidden (or ‘latent’) states usually represent the behaviour of the animal and are thought to drive the movement (e.g. lower speed when foraging; Langrock *et al.*, 2012). The state process of HMMs generally assumes that the probability of being in a particular state (behaviour) at time *t*, *X_t_*, is dependent only on the state at time *t-1*. This dependence is encoded in the transition probability matrix, which contains the probability of switching between each behavioural state (each referred as a transition probability). The link between the behavioural state and movement is represented by the emission probabilities, which model each observation, *Y_t_*, as a function of the state at that time (Langrock *et al.*, 2012; McClintock *et al.*, 2020). Our HMM used the five data streams described above: step lengths (in metres), turning angles (in radians), maximum dive depths (in metres), dive wiggles (in metres) and distance to shore (in kilometres). We modelled step lengths, maximum dive depths, dive wiggles and distance to shore using a gamma distribution given they were non-zero positive values. Turning angles were modelled using a von Mises distribution to handle the circular nature of angles.

**Figure 1:**
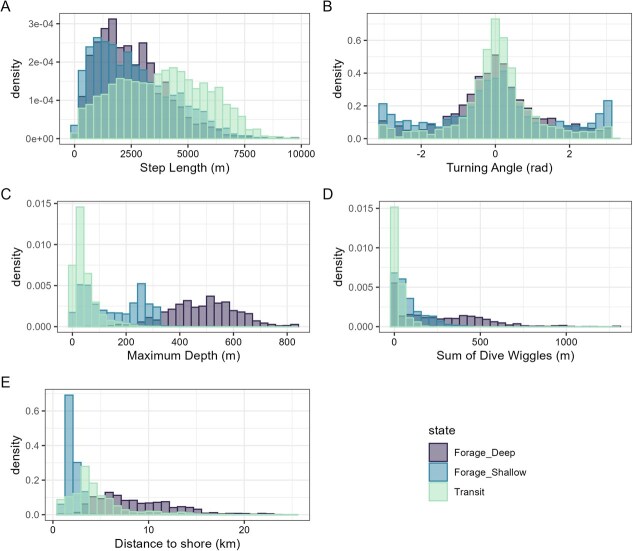
Distributions for the five data streams describing movement behaviour of narwhal in Eclipse Sound. Data streams included step length and turning angle for each location as well as maximum depth, the sum of dive wiggles for diving behaviour and distance to shore. These data streams were separated into three behavioural states via an HMM, including foraging deep, foraging shallow and transiting states.

The composite stress index highlights the impact that relative, compounding stressors can have on individual movement decisions and was included as a covariate acting on both transition probabilities and on the emission probabilities of step lengths, maximum dive depths and distance to shore. Specifically, we included the index as a covariate in the transition probabilities between different states using a logit transformation to ensure that the probability values remained between 0 and 1 and in the mean of the emission distributions for each state for the three data streams using a log transformation to ensure that the mean remained positive using the transition formula and pseudo-design matrices provided in ‘momentuHMM’ (see McClintock and Michelot, 2018). By including the composite stress index as a covariate in the transition probability, it can modify the probability of switching between each state, thus allowing it to affect the behavioural budget of the animals. By including it as a covariate in the mean of the emission probabilities, it can modify how fast, deep and far from shore the narwhal moves in each state. We did not include any diurnal effects in this model as the season in which these data were collected only included daylight and civil twilight. Parameters of the emission distributions and transition probabilities were estimated using the R package ‘momentuHMM’ (McClintock and Michelot, 2018). Model-fitting algorithms were informed by starting values derived from fits by individual HMMs for each data stream separately. Individual state sequences were decoded using the Viterbi algorithm to derive the most likely sequence of behaviours (Zucchini and MacDonald, 2009). The number of behaviour states and candidate models were selected by following typical model selection methods for HMMs (Pohle *et al.*, 2017).

## Results

**Figure 2:**
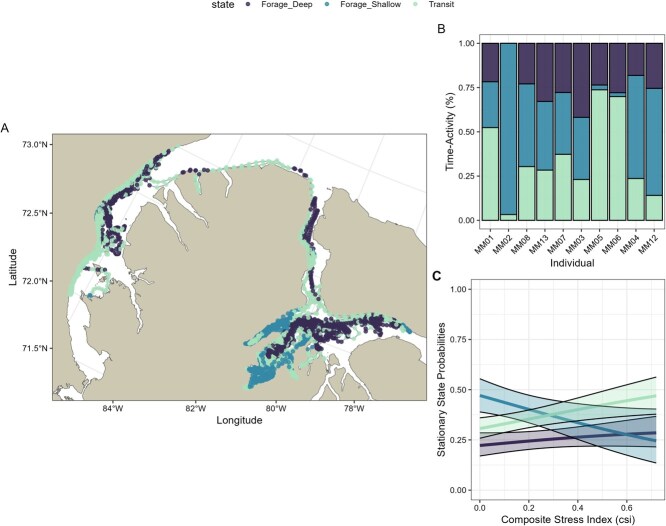
Stress index impact behavioural state probabilities. Behavioural states were decoded using the Viterbi algorithm as the most likely state. (**A**) Map of locations coloured according to behavioural state, and (**B**) the percentage of time over a month that individuals spent in each of the three behavioural states, with individuals ordered by increasing composite stress index. Stationary state probabilities (**C**) across composite stress index (CSI) scores for three behavioural states in narwhal (foraging deep, foraging shallow and transiting behaviours). Shaded regions around each line represent the 95% confidence intervals.

Composite stress index values were determined for all 10 narwhal with GPS-enabled location data and dive behaviour (Table 1). From these data, three behavioural states were derived from our HMM (Fig. 1). The first behaviour, ‘transiting’, was described by little diving (maximum depth: 46.4 m; dive wiggles: 26.6 m) and directed movement (step length: 3832.04; angle concentration: 1.57). The distance to shore associated with transiting was variable (4.58 ± 2.8 km) but occurred at an intermediate distance to shore when compared to the two other behavioural states. The other two states differed primarily in their dive depths, dive wiggles and distance to shore. ‘Foraging shallow’ was characterized by shorter step lengths (mean 2587 m) as well as shallower dives (mean 153 m) and dive wiggles (89.5 m), while largely taking place in the nearshore (mean 2.12 km). ‘Foraging deep’ included small step lengths (mean 2528.5 m), deep dives (mean 481.5 m) and greater dive wiggles (mean 327.3 m), while in the offshore (mean 8.7 km). Viterbi-decoded behavioural states for each narwhal track are shown in Fig. 2A, as well as the resulting differences in time activity budgets (Fig. 2B). Full model results and model selection can be found in the Supplementary Materials.

The stationary state probabilities for each behavioural state as a function of composite stress index can be found in Fig. 2C. Narwhal with a higher relative stress index were found to prioritize transiting over foraging behaviours as compared to narwhal with a lower relative stress index.

The composite stress index appeared to modulate transition probabilities between behavioural states. Animals with a higher stress index were more likely to transition from either foraging state (foraging shallow or foraging deep) to a transiting state than those with a lower stress index. Likewise, individuals with a higher stress index were also likely to transition to a foraging shallow behaviour from the other two behavioural states. All other regression coefficients on transition probabilities overlapped with zero (see Table S3).

**Figure 3:**
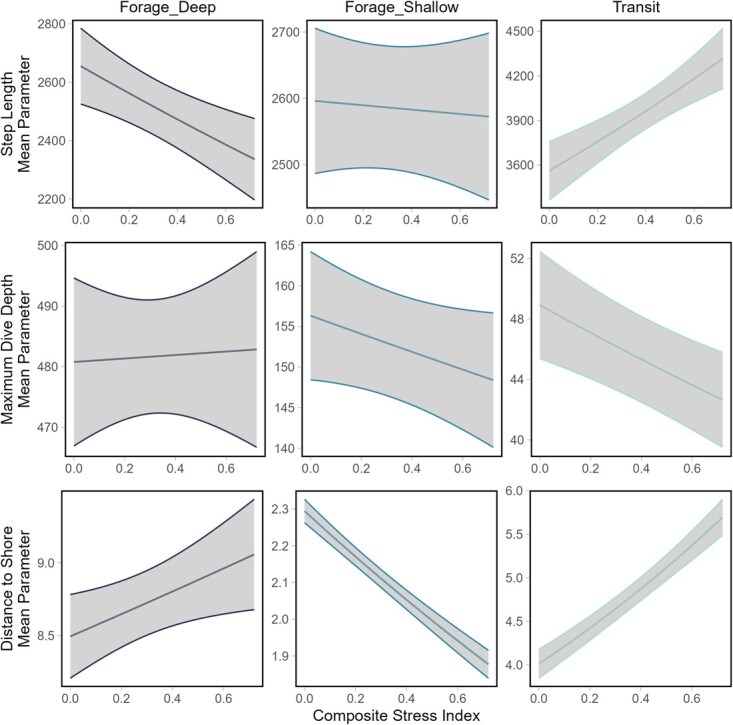
Stress mediates emission probabilities of observed behaviours. Composite stress index was included as a covariate on the emission probabilities of five data streams (step length, turning angle, maximum dive depth, sum of dive wiggles and distance to shore) used to characterize three behavioural states (foraging deep, foraging shallow and transiting).

The composite stress index was also found to modulate the emission probabilities of step length, dive depth and distance to shore of each behavioural state (Fig. 3, Table S4). Animals with a higher composite stress index score appeared to take shorter steps and were positioned further offshore when in a foraging deep state than individuals with a lower stress index score. Individuals with a higher composite stress index were also found to be closer to shore in a foraging shallow behaviour than individuals with a lower stress index. Animals with a higher composite stress index engaging in transiting behaviour took longer steps on average, had shallower dives (maximum depth) and were further offshore than animals with a lower stress index.

We explored alternative model formulations and composite stress indices to further explore the influence that the composite stress index had on aspects of movement as well as the influence of missing data on index values, respectively, which are detailed in the Supplementary Materials. First, we compared the full model to models with no covariate effects, composite stress index effects only on the transition probabilities and stress index effects only on emission probabilities. The full model was found to have the lowest AIC as compared all four models. The second best model was the model with stress index effects only on emission probabilities (ΔAIC = 37.0). Second, we explored an alternative composite stress index utilizing only complete cases of variables measured across all individuals to assess the influence of missing data on our conclusions. We found little change in parameters between these two stress index formulations.

## Discussion

Through integrating information gathered from health assessments and stress indices, we demonstrate the utility of including a composite stress index as a covariate in HMM analyses of movement ecology. Our composite stress index highlighted subtle yet potentially important changes in behavioural priorities for individual narwhal due to variations in the effects of various stressors and health markers. Narwhal with a greater composite stress index score were found to prioritize transiting over diving behaviours (Fig. 2C) and displayed significant differences in the expression of each behavioural state, specifically differences in step lengths and maximum dive depths (Fig. 3). The composite stress index was also able to capture distinct differences in behaviours relative to distance to shore (Fig. 3).

Narwhal are deep-diving specialists, often found diving to great depths to reach preferred prey (Williams *et al.*, 2011; Watt *et al.*, 2015). While it is difficult to directly evaluate if the changes in behaviour noted here correspond to true foraging opportunities (see Florko *et al.*, 2023), our data suggest that individuals with a higher composite stress index may spend less time in either of the foraging states (i.e. shallow foraging and deep foraging) in favour of transiting than narwhals with a lower composite stress index. A loss of foraging activity, either because of shallower dives (as characterized by emission probabilities) or less time spent in a foraging state than transiting, may result in less time accessing preferred foraging locations. In other whale species, changes in behaviour as a result of acute disturbance were found to disrupt feeding opportunities (DeRuiter *et al.*, 2017), while compromised individuals saw decreases in dive depths and lowered overall condition (Holser *et al.*, 2023). Other species experiencing marked changes in activity budgets were also noted to have marked increases in energy requirements from altered behaviour, suggesting allostatic overload (Arlettaz *et al.*, 2015). Our method captures similar changes in behavioural priorities to those previously found for narwhal in response to acute stressors such as shipping noise (Tervo *et al.*, 2021; Tervo *et al.*, 2023), seismic testing (Heide-Jørgensen *et al.*, 2021; Williams *et al.*, 2022), capture and handling (Shuert *et al.*, 2021) and predators (Laidre *et al.*, 2006; Breed *et al.*, 2017), which sometimes lasted well beyond the immediate stimulus. The specific consequences of changes in narwhal behaviour to these stressors have been extensively reported elsewhere (i.e. Heide-Jørgensen *et al.*, 2021; Tervo *et al.*, 2023). Importantly, our results highlight how incorporating allostatic load indices as a covariate in movement models can explain some of the commonly found, but generally unknown inter-individual variance in movement behaviour (Dingemanse *et al.*, 2022; Stuber *et al.*, 2022; Wong *et al.*, 2022).

Modelling the effects of cumulative, long-term stress on movement ecology have classically been very difficult to achieve. In well-studied species in the wild, new modelling techniques such as population consequences of disturbance (e.g. PCoD models; Pirotta *et al.*, 2018) allow researchers to understand how stressors may impact vital rates such as calving rates or mortality. These models primarily rely on well-characterized mechanistic relationships between animal responses to known stressors and forecast these acute and chronic effects towards vital rates, making them invaluable for risk assessments (Keen *et al.*, 2021). Studies on humans and animals in managed care have seen success in developing and evaluating allostatic load indices measured as single time points as a utilitarian biomarker of the effects of sub-clinical cumulative stress (Edes *et al.*, 2021; Seeley *et al.*, 2022), but few efforts have attempted to quantify this in a fully wild context. Here, we attempted to quantify and understand the impact of multiple additive stress markers on the movement ecology of narwhal. Stressors evaluated here within the composite stress index represent multiple markers of potential compounding effects from increasing stress load (Edes *et al.*, 2018), and as a result are largely non-specific to a single stimulus, disease or other specific stress source. Our composite stress index here, as with other allostatic load indices, is not intended to diagnose or capture specific health conditions. Instead, our composite stress index aims to quantify how the culmination of a variety of non-specific health parameters, such as the burden of lactation energy costs (Fedak and Anderson, 1982), elevated stress (Gundlach *et al.*, 2018) and parasites (Schick *et al.*, 2013; Pirotta *et al.*, 2023), would result in relative differences of total stress load between individuals. The fact that relative differences in narwhal stress index scores linked directly to differences in activity budgets and behavioural priorities highlights that the inclusion of health markers, and their variability, may be important variables to consider in more complex movement modelling efforts and risk assessments.

Individuals may react differently to stressors; some individuals may have reaction norms that absorb some of a stress response effectively (e.g. not marked as positive in our stress index), while others may respond more strongly to stress. These individual differences in stress responses are most often discussed in aspects of reactive scope models (Romero *et al.*, 2009) and elsewhere in dimensions of stress-coping styles (e.g. Koolhaas *et al.*, 2010; Costantini *et al.*, 2012). Our exploration of relative, compounding stressors and the development of this index therein provides a means to explore the way that stress responses within an individual may dictate behaviour, rather than a distinct set of clinical thresholds. Even when we only considered a limited number of stress markers measured across all individuals (Supplementary Materials), we observed similar effects in our models, further highlighting that even a few markers may be informative in elucidating relative differences in stress and its effects on movement behaviour.

Individual variation in movement can also result from other aspects of animal behaviour (i.e. personality type; Spiegel *et al.*, 2017) that are commonly unobserved factors in telemetry data. For example, narwhal in this study showed marked variability in the percentage of time spent in each behaviour (Fig. 2B). McClintock (2021) argued that covariates, like a composite stress index, are the preferred route to develop an understanding of individual heterogeneity in movement ecology analyses. Including random effects to model the transition probabilities (as in McClintock, 2021) could potentially disentangle any remaining individual variability from the effect of the composite stress index. While our sample size was too small to include both a composite stress index and random effects in the model (McClintock, 2021), studies with a larger sample size should consider modelling the transition between behaviours with both the composite stress index and random effects.

Health assessments of wildlife allow understanding of what makes an individual healthy (Kophamel *et al.*, 2021), but can also reveal early warning indicators of ecosystem stress and human health impacts (Dudley *et al.*, 2015; Ruscio *et al.*, 2015). While some species benefit from concerted efforts to track and gather animal health data (Barratclough *et al.*, 2019), many Arctic species are difficult to access resulting in data deficiencies. Such a limited sample size can mean that evaluating critical questions over the health of Arctic marine mammals becomes even harder to detect (Sequeira *et al.*, 2019). By incorporating a flexible approach to information inclusion, the composite stress index was able to maximize the amount of usable information from each individual narwhal, extending the allostatic load index approach. Future work could aim to incorporate more accurate but more computationally expensive methods for handling missing data. For example, multiple imputation has been successfully applied to larger datasets for assessing allostatic load in human health (Robertson *et al.*, 2017; Obeng-Gyasi *et al.*, 2023) and could be incorporated into the HMM framework (e.g. McClintock, 2017). With a greater understanding of the underlying dynamic resource landscape of the region, more advanced spatial models using recharge dynamics (i.e. parameters to factor in energy gained from the environment) could be used to elucidate how composite stress indices are linked to individual variation in fine-scale decisions (Hooten *et al.*, 2019). While we were only able to assess changes in movement ecology in a small number of individuals due to sample size limitations, we argue that the inclusion of this ancillary information gathered during health assessments may be critical to informing movement ecology models going forward.

Many of the parameters included in our composite stress index were borrowed from large whales, where data must be gathered solely from observations (Schick *et al.*, 2013; Pirotta *et al.*, 2023). New techniques for measuring health remotely through the collection of samples from drone-based photogrammetry (i.e. body condition; Christiansen *et al.*, 2020; Fearnbach *et al.*, 2018; Shero *et al.*, 2021) or blow samples (i.e. microbiomes; Apprill *et al.*, 2017; Atkinson *et al.*, 2021) can provide a wealth of information without the need to handle animals. Even when not specific to direct stressors, drone-based photogrammetry, for example, has shown promise for evaluating population-specific differences in animal size associated with niche separation (Bierlich *et al.*, 2023). Incorporating these types of information within an allostatic load framework may also explain variation in animal movement. These new techniques extend the applicability of incorporating a composite stress index in standard animal movement analyses. However, it is important to acknowledge that more work is necessary to validate our composite stress index, such as through studies of individuals in managed care (Beer *et al.*, 2023), where a full suite of lifelong stressors can be assessed and compared to the subset of metrics combined in the composite stress index presented here. While single-point measures of animal health have shown promise in explaining long-term survival (Festa-Bianchet *et al.*, 1997; Hall *et al.*, 2002; Shuert *et al.*, 2015), extending the allostatic load index approach here also highlights that single-point measures of animal health can inform on short-term animal behaviour.

With the Arctic becoming more accessible, the presence of anthropogenic stressors such as shipping noise (Dawson *et al.*, 2018) and natural resource extraction continue to rise in areas previously receiving few visitors. While some progress has been made in our understanding of the impact of anthropogenic stressors on narwhal (Tervo *et al.*, 2023), our approach captured expected behaviours previously observed for this species and improved model performance for determining behavioural priorities and expression. This finding underscores the utility of incorporating relative stress indices in movement ecology modelling. Many of the stress and health parameters used in our composite stress index are usually collected simultaneously during tagging and handling events (Goertz *et al.*, 2019; Kophamel *et al.*, 2021), but are often analysed as separate research priorities. Given the wealth of health and stress information likely already collected, we argue that allostatic load indices in movement modelling represents an attainable goal to understand relative health impacts on movement behaviour for a multitude of aquatic and terrestrial species.

## Supplementary Material

Web_Material_coaf022

## Data Availability

Data from this manuscript are available from the authors upon reasonable request.
