# Supplementary material for: Putting the health in hidden Markov models: incorporating allostatic load indices into movement ecology analyses
Source: Conserv Physiol. 2025 Apr 11;13(1):coaf022. doi: 10.1093/conphys/coaf022 (PMC11991693; doi:10.1093/conphys/coaf022)
Supplement: Web_Material_coaf022 [file web_material_coaf022.zip › CONPHYS-2024-048.R2_SUPP_clean .pdf]

Title: "Putting the Health in Hidden Markov Models: Incorporating allostatic stress load indices into movement ecology analyses"

Authors: Courtney R. Shuert[1,2,*], Marie Auger-Méthé[3,4], Karine Béland[5], Nigel E. Hussey[6], Marion R. Desmarchelier[5], Marianne Marcoux[1]

[1]Arctic and Aquatic Research Division, Freshwater Institute, Fisheries and Oceans Canada
[2]Conservation and Research, Assiniboine Park Conservancy
[3]Department of Statistics, University of British Columbia
[4]Institute for the Oceans & Fisheries, University of British Columbia
[5]Department of Clinical Sciences, Faculté de Médecine Vétérinaire, Université de Montréal
[6]Department of Biosciences, University of Windsor

Supplementary Materials

Alternative Model Formulations

Here, we present model results for alternative model formulations for a 3-state hidden Markov model to evaluate movement and stress responses in narwhal, including 1) the full model included in the main text with the composite stress index covariate acting on both the transition and emission probabilities within a 3-state model ('Original Model', AIC = 220187.2), 2) emission probability covariate effects only ('Emission Only,' AIC = 220225.1), 3) a 3-state model with transition probability covariate effects only ('Transition Only', AIC = 220551.0), and 4) a 3-state model no covariate effects included in the model ('No Covariate Effects', AIC = 220412.4). Given the information provided by these alternative model formulations (Tables S1-S4) and subsequent AIC rankings, it appears that the composite stress index had the greatest effect overall on emission probability distributions. As a result, we did not see marked difference in activity budgets between model formulations (Figure S1).

Figure S1: Activity Budgets for Alternative Models. Activity budgets generated from alternative model formulations for describing the effects of composite stress index on behaviour of narwhals. Models include the model presented in the main text (Original Model), no covariate effects (No Covariates), covariate effects on emission probabilities only (Emission Only), and covariate effects on transition probabilities only (Transition Only).

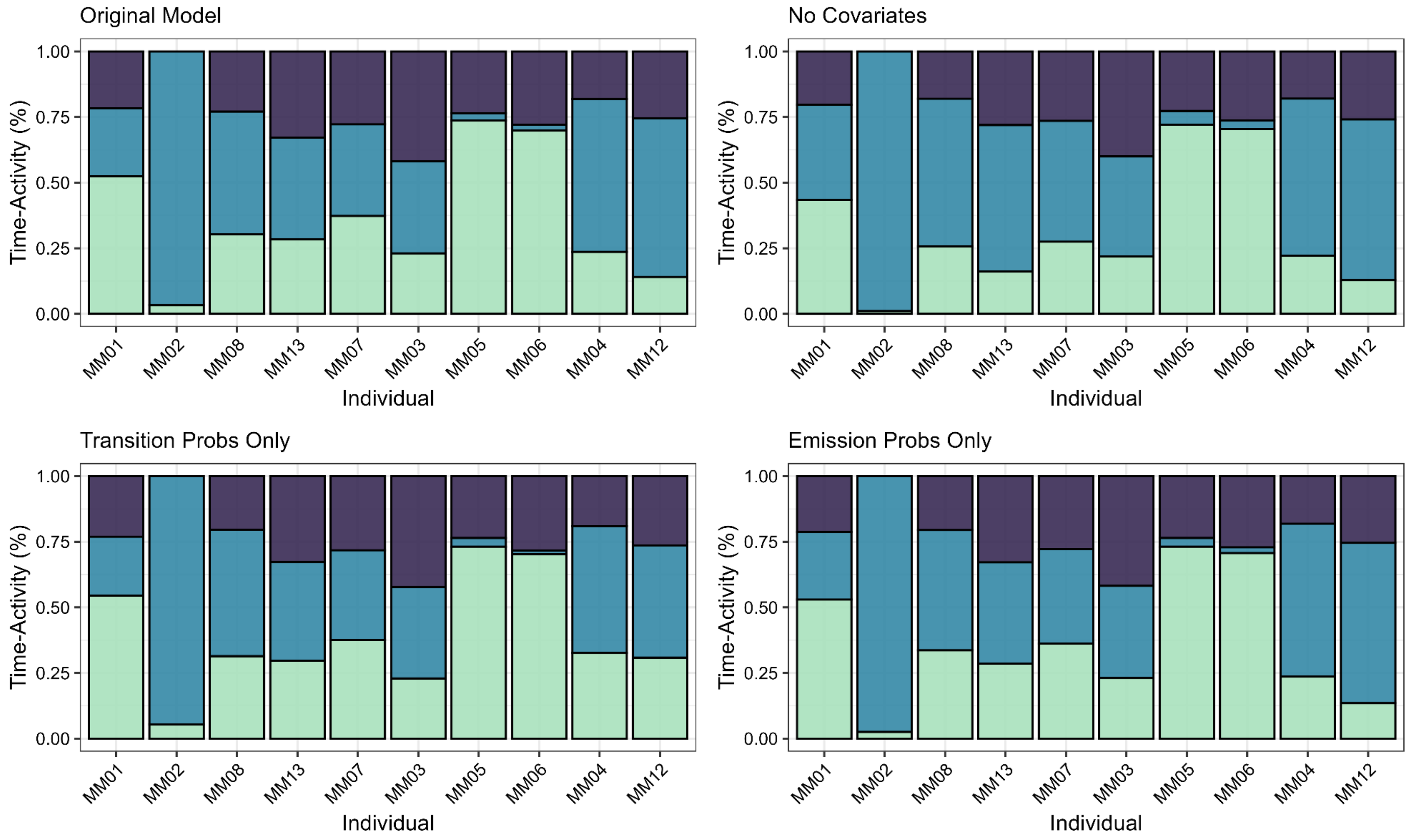

Table S1: Data stream estimates based on mean covariate value for all model formulations. Mean data stream estimates (standard deviation) for each behavioural state derived from HMM. In order to handle a few timesteps where no information on dive wiggles was available (zero inflation), an additional zero-mass parameter is included. Included are estimates from the original model, those including covariate effects on the emission probabilities only, transition probabilities only, and a model with no covariate effects.

	(Emission and Transition - Original)			(Emission Only)		
	Forage_Deep	Forage_Shallow	Transit	Forage_Deep	Forage_Shallow	Transit
Step Length (m)	2528.52<br>1564.79	2587.07<br>1870.66	3832.04<br>2305.47	2526.27<br>1568.79	2584.9<br>1866.61	3837.2<br>2314.77
Turning Angle (rad)	0<br>1.079	0<br>0.487	0<br>1.572	0<br>1.078	0<br>0.488	0<br>1.572
Max Depth (m)	481.56<br>136.92	153.24<br>126.82	46.42<br>39.63	481.66<br>137.005	152.97<br>127.315	46.79<br>39.96
Dive Wiggles (m)	327.39<br>(263.24, 0.215)	89.53<br>(88.36, 0.089)	26.67<br>(27.60, 0.130)	327.8<br>(263.02, 0.215)	89.26<br>(88.43, 0.090)	26.97<br>(27.95, 0.129)
Distance to Shore (km)	8.7<br>4.1	2.12<br>0.53	4.58<br>2.79	8.72<br>4.09	2.12<br>0.53	4.59<br>2.79
	(Transition Only)			(No Covariate Effects)		
	Forage_Deep	Forage_Shallow	Transit	Forage_Deep	Forage_Shallow	Transit
Step Length (m)	2555.43<br>1600.73	2599.34<br>1833.21	3739.38<br>2426.18	2431.88<br>1523.39	2640.64<br>1921.23	4091.58<br>2233.34
Turning Angle (rad)	0<br>1.097	0<br>0.488	0<br>1.421	0<br>1.031	0<br>0.533	0<br>1.837
Max Depth (m)	475.69<br>147.34	167.49<br>128.9	43.277<br>35.67	489.82<br>125.61	140.18<br>124.48	50.96<br>46.8
Dive Wiggles (m)	322.39<br>(261.73, 0.212)	97.48<br>(90.79, 0.089)	24.48<br>(24.38, 0.127)	336.05<br>(263.98, 0.220)	82.22<br>(85.35, 0.095)	30.59<br>(33.00, 0.127)
Distance to Shore (km)	8.7<br>4.092	2.2<br>0.49	4.36<br>2.87	8.87<br>4.1	2.1<br>0.66	5.34<br>2.74

Table S2: Transition Probability Matrix. Estimated transition probabilities between each of the three behavioural states based on mean covariate values.

(Original Model)

	Forage Deep	Forage Shallow	Transit
Forage Deep	0.851	0.002	0.146
Forage Shallow	0.002	0.962	0.035
Transit	0.098	0.036	0.865

(Emission Only)

	Forage Deep	Forage Shallow	Transit
Forage Deep	0.847	0.002	0.150
Forage Shallow	0.002	0.963	0.034
Transit	0.100	0.037	0.862

(Transition Only)

	Forage Deep	Forage Shallow	Transit
Forage Deep	0.854	0.002	0.143
Forage Shallow	0.002	0.944	0.052
Transit	0.089	0.046	0.863

(No Covariate Effects)

	Forage Deep	Forage Shallow	Transit
Forage Deep	0.836	0.002	0.161
Forage Shallow	0.002	0.974	0.022
Transit	0.115	0.034	0.849

Table S3: Regression coefficients on transition probabilities. Composite stress index was included as a covariate on transition probabilities between three behavioural states: (1) Foraging deep, (2) foraging shallow, and (3) transiting presented on the working scale. Covariate effects significantly different from zero are highlighted in bold.

	(Original Model)		(Transition Only)	
Transition	Intercept	CSI	Intercept	CSI
Forage deep → forage shallow	-6.000<br>(-8.341, -3.659)	0.182<br>(-5.488, 5.853)	-5.769<br>(-7.600, -3.937)	-0.735<br>(-5.927, 4.502)
Forage deep → transit	-1.993<br>(-2.230, -1.755)	**0.847**<br>**(0.295, 1.399)**	-1.95<br>(-2.184, 0.067)	**0.611**<br>**(0.067, 1.155)**
Forage shallow → forage deep	-5.809<br>(-6.942, -4.675)	-0.978<br>(-4.562, 2.604)	-5.838<br>(-6.936, -4.741)	0.123<br>(-2.838, 3.084)
Forage shallow → transit	-2.991<br>(-3.272, -2.710)	**-1.175**<br>**(-2.021, -0.329)**	-2.832<br>(-3.104, -2.559)	-0.209<br>(-0.924, 0.504)
Transit → forage deep	-2.303<br>(-2.553, -2.052)	0.476<br>(-0.091, 1.043)	-2.269<br>(-2.511, -2.027)	0.027<br>(-0.513, 0.568)
Transit → forage shallow	-2.402<br>(-2.707, -2.096)	**-3.862**<br>**(-3.862, -1.753)**	-2.338<br>(-2.625, -2.051)	**-2.115**<br>**(-2.952, -1.277)**

Table S4: Model parameter estimates of emission probabilities. Composite stress index (CSI) was included as a covariate on step length, maximum dive depth, and distance to shore presented on the working scale. All others represent model parameter estimates for each data stream without covariate effects. Parameter estimates are included, with 95% confidence intervals included below in each in brackets. Significant effects (confidence intervals do not overlap zero) of CSI as a covariate are highlighted in bold.

		(Original)			(Emission Only)		
		Forage_Deep	Forage_Shallow	Transit	Forage_Deep	Forage_Shallow	Transit
Step Length	Intercept	7.888<br>(7.835, 7.933)	7.861<br>(7.819, 7.903)	8.177<br>(8.122, 8.233)	7.883<br>(7.834, 7.933)	7.86<br>(7.817, 7.903)	8.178<br>(8.120, 8.237)
	**CSI**	**-0.177**<br>**(-0.284, -0.071)**	-0.012<br>(-0.090, 0.064)	**0.267**<br>**(0.153, 0.381)**	**-0.179**<br>**(-0.286, -0.073)**	-0.01<br>(-0.088, 0.068)	**0.268**<br>**(0.146, 0.391)**
	sd	7.36<br>(7.306, 7.415)	7.532<br>(7.486, 7.578)	7.747<br>(7.702, 7.791)	7.358<br>(7.302, 7.413)	7.531<br>(7.485, 7.578)	7.747<br>(7.701, 7.792)
Angle	Concentration	0.076<br>(-0.018, 0.171)	-0.718<br>(-0.861, -0.574)	0.452<br>(0.378, 0.527)	0.075<br>(-0.020, 0.170)	-0.715<br>(-0.857, -0.572)	0.452<br>(0.377, 0.527)
Max Depth	Intercept	6.175<br>(6.146, 6.204)	5.051<br>(5.001, 5.102)	3.889<br>(3.817, 3.962)	6.175<br>(6.146, 6.204)	5.046<br>(4.996, 5.096)	3.9<br>(3.827, 3.974)
	**CSI**	0.005<br>(-0.006, 0.073)	-0.072<br>(-0.160, 0.016)	**-0.19**<br>**(-0.295, -0.085)**	0.005<br>(-0.062, 0.073)	-0.059<br>(-0.147, 0.028)	**-0.201**<br>**(-0.306, -0.095)**
	sd	4.919<br>(4.847, 4.990)	4.842<br>(4.791, 4.893)	3.679<br>(3.593, 3.765)	4.92<br>(4.844, 4.995)	4.846<br>(4.795, 4.898)	3.688<br>(3.599, 3.776)
Dive Wiggles	Intercept	5.791<br>(5.734, 5.847)	4.494<br>(4.441, 4.547)	3.283<br>(3.202, 3.365)	5.792<br>(5.735, 5.849)	4.491<br>(4.438, 4.544)	3.295<br>(3.211, 3.378)
	sd	5.573<br>(5.501, 5.644)	4.481<br>(4.420, 4.542)	3.318<br>(3.218, 3.417)	5.572<br>(5.501, 5.643)	4.482<br>(4.421, 4.542)	3.33<br>(3.227, 3.433)
	zero mass	-1.292<br>(-1.441, -1.143)	-2.314<br>(-2.490, -2.139)	-1.894<br>(-2.061, -1.728)	-1.29<br>(-1.440, -1.141)	-2.307<br>(-2.481, -2.134)	-1.901<br>(-2.067, -1.735)
Distance to Shore	Intercept	2.139<br>(2.105, 2.173)	0.83<br>(0.816, 0.844)	1.388<br>(1.348, 1.429)	2.141<br>(2.108, 2.175)	0.83<br>(0.816, 0.845)	1.391<br>(1.349, 1.433)

	**CSI**	**0.089 (0.011, 0.166)**	**-0.255 (-0.288, -0.221)**	**0.486 (0.411, 0.562)**	**0.088 (0.010, 0.166)**	**-0.278 (-0.313, -0.242)**	**0.483 (0.406, 0.560)**
	sd	1.411 (1.369, 1.454)	-0.623 (-0.660, -0.586)	1.027 (0.985, 1.068)	1.41 (1.367, 1.453)	-0.618 (-0.657, -0.578)	1.026 (0.985, 1.067)
			(Transition Only)			(No Covariate Effects)	
		Forage_Deep	Forage_Shallow	Transit	Forage_Deep	Forage_Shallow	Transit
Step Length	Intercept	7.845 (7.808, 7.883)	7.863 (7.827, 7.898)	8.226 (8.195, 8.257)	7.796 (7.757, 7.835)	7.878 (7.845, 7.911)	8.316 (8.286, 8.346)
	sd	7.378 (7.324, 7.432)	7.513 (7.466, 7.561)	7.794 (7.751, 7.836)	7.328 (7.271, 7.385)	7.56 (7.517, 7.604)	7.711 (7.664, 7.757)
Angle	Concentration	0.093 (-0.000, 0.186)	-0.715 (-0.864, -0.566)	0.351 (0.282, 0.420)	0.031 (-0.068, 0.131)	-0.628 (-0.748, -0.509)	0.608 (0.537, 0.679)
Max Depth	Intercept	6.164 (6.144, 6.185)	5.12 (5.076, 5.165)	3.767 (3.715, 3.819)	6.194 (6.176, 6.211)	4.942 (4.901, 4.983)	3.931 (3.858, 4.003)
	sd	4.992 (4.929, 5.056)	4.859 (4.806, 4.911)	3.574 (3.502, 3.646)	4.833 (4.773, 4.892)	4.824 (4.774, 4.874)	3.846 (3.747, 3.944)
Dive Wiggles	Intercept	5.775 (5.719, 5.832)	4.579 (4.525, 4.634)	3.197 (3.127, 3.268)	5.817 (5.760, 5.873)	4.409 (4.358, 4.460)	3.42 (3.340, 3.501)
	sd	5.567 (5.496, 5.638)	4.508 (4.446, 4.570)	3.194 (3.108, 3.280)	5.575 (5.503, 5.647)	4.446 (4.387, 4.506)	3.496 (3.401, 3.591)
	zero mass	-1.312 (-1.461, -1.164)	-2.32 (-2.506, -2.133)	-1.921 (-2.080, -1.763)	-1.262 (-1.412, -1.111)	-2.248 (-2.406, -2.090)	-1.921 (-2.101, -1.742)
Distance to Shore	Intercept	2.164 (2.138, 2.189)	0.792 (0.781, 0.803)	1.474 (1.444, 1.503)	2.182 (2.156, 2.209)	0.741 (0.729, 0.754)	1.676 (1.649, 1.702)
	sd	1.409 (1.367, 1.450)	-0.709 (-0.748, -0.669)	1.055 (1.014, 1.096)	1.413 (1.363, 1.462)	-0.407 (-0.440, -0.373)	1.011 (0.969, 1.053)

Alternative Composite Stress Index

We explored an alternative definition of the composite stress index using only the parameters for which we had information for all 10 narwhals. This included the first three parameters and associated weights found in Table 1: The presence of healed scars, the presence of active wounds, and the presence of ectoparasites. A comparison between the two composite stress index rankings is highlighted in Figure S2. In all, we found very similar behavioural responses using the smaller composite stress index as compared to those presented in the main text and an overall higher AIC value (220299.7). Tables S5-S8 highlight comparisons between the parameter estimates for the original model (found in the main text) to that using the covariates of complete cases only.

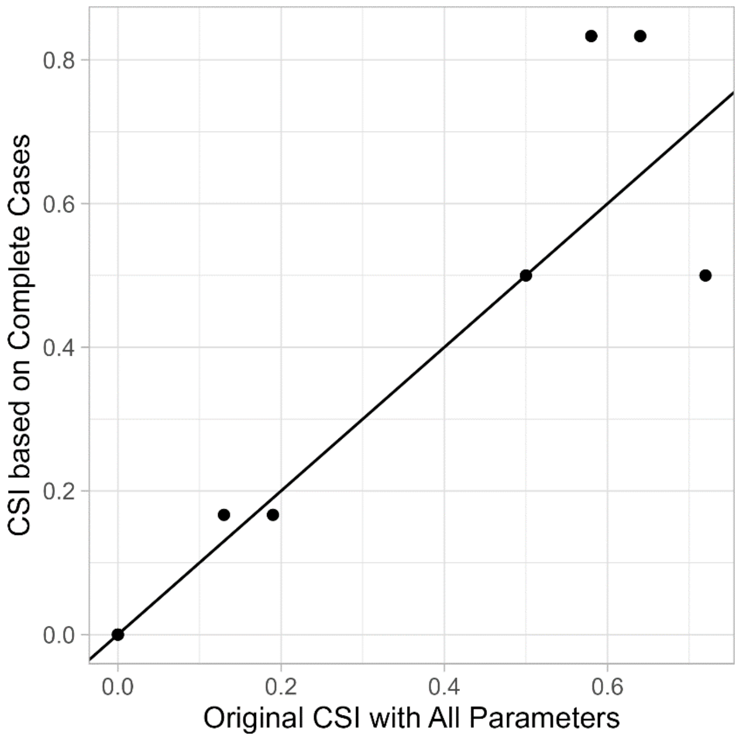

Figure S2: Comparison of composite stress index (CSI) rankings based on all covariates against a CSI based only on three parameters for which we had information from all whales (complete cases, including healed scars, active wounds, and ectoparasites).

Table S5: Data stream estimates based on mean covariate value for alternative CSI formulations. Mean data stream estimates (standard deviation) for each behavioural state derived from HMM. In order to handle a few timesteps where no information on dive wiggles was available (zero inflation), an additional zero-mass parameter is included.

	(Orignal CSI)			(CSI Complete Cases Only)		
	Forage_Deep	Forage_Shallow	Transit	Forage_Deep	Forage_Shallow	Transit
Step Length (m)	2528.52<br>1564.79	2587.07<br>1870.66	3832.04<br>2305.47	2494.98<br>1549.12	2588.90<br>1890.39	3888.61<br>2273.9
Turning Angle (rad)	0<br>1.079	0<br>0.487	0<br>1.572	0<br>1.063	0<br>0.484	0<br>1.659
Max Depth (m)	481.56<br>136.92	153.24<br>126.82	46.42<br>39.63	484.20<br>135.27	152.17<br>127.57	46.71<br>40.08
Dive Wiggles (m)	327.39<br>(263.24, 0.215)	89.53<br>(88.36, 0.089)	26.67<br>(27.60, 0.130)	326.36<br>(262.89, 0.217)	88.98<br>(88.70, 0.091)	27.01<br>(28.11, 0.129)
Distance to Shore (km)	8.70<br>4.10	2.12<br>0.53	4.58<br>2.79	8.75<br>4.00	2.10<br>0.57	4.66<br>2.82

Table S6: Transition Probability Matrix. Estimated transition probabilities between each of the three behavioural states based on mean covariate values.

(Original Model)

	Forage Deep	Forage Shallow	Transit
Forage Deep	0.851	0.002	0.146
Forage Shallow	0.002	0.962	0.035
Transit	0.098	0.036	0.865

(CSI Complete Cases only)

	Forage Deep	Forage Shallow	Transit
Forage Deep	0.857	0.002	0.140
Forage Shallow	0.002	0.961	0.036
Transit	0.098	0.041	0.859

Table S7: Regression coefficients on transition probabilities. Composite stress index was included as a covariate on transition probabilities between three behavioural states: (1) Foraging deep, (2) foraging shallow, and (3) transiting presented on the working scale. Covariate effects significantly different from zero are highlighted in bold.

	(Original Model)		(CSI Complete Cases only)	
Transition	Intercept	CSI	Intercept	CSI
Forage deep → forage shallow	-6.000<br>(-8.341, -3.659)	0.182<br>(-5.488, 5.853)	-6.526<br>(-0.650, -3.401)	1.596<br>(-3.651, 6.844)
Forage deep → transit	-1.993<br>(-2.230, -1.755)	**0.847**<br>**(0.295, 1.399)**	-2.132<br>(-2.372, -1.893)	**1.102**<br>**(0.637, 1.566)**
Forage shallow → forage deep	-5.809<br>(-6.942, -4.675)	-0.978<br>(-4.562, 2.604)	-5.942<br>(-7.126, -4.758)	-0.214<br>(-3.286, 2.856)
Forage shallow → transit	-2.991<br>(-3.272, -2.710)	**-1.175**<br>**(-2.021, -0.329)**	-3.131<br>(-3.422, -2.838)	-0.508<br>(-1.277, 0.260)
Transit → forage deep	-2.303<br>(-2.553, -2.052)	0.476<br>(-0.091, 1.043)	-2.26<br>(-2.503, -2.016)	0.307<br>(-0.135, 0.749)
Transit → forage shallow	-2.402<br>(-2.707, -2.096)	**-3.862**<br>**(-3.862, -1.753)**	-2.391<br>(-2.699, -2.084)	**-2.134**<br>**(-2.983, -1.284)**

Table S8: Model parameter estimates of emission probabilities. Composite stress index (CSI) was included as a covariate on step length, maximum dive depth, and distance to shore as presented on the working scale. All others represent model parameter estimates for each data stream without covariate effects. Parameter estimates are included, with 95% confidence intervals included below in each in brackets. Significant effects (confidence intervals do not overlap zero) of CSI as a covariate are highlighted in bold.

		(Original CSI)			(CSI Complete Cases only)		
		Forage_Deep	Forage_Shallow	Transit	Forage_Deep	Forage_Shallow	Transit
Step Length	Intercept	7.888	7.861	8.177	7.860	7.860	8.215
		(7.835, 7.933)	(7.819, 7.903)	(8.122, 8.233)	(7.812, 7.907)	(7.820, 7.899)	(8.172, 8.259)
	**CSI**	**-0.177**	-0.012	**0.267**	**-0.127**	-0.004	**0.168**
		**(-0.284, -0.071)**	(-0.090, 0.064)	**(0.153, 0.381)**	**(-0.218, -0.037)**	(-0.075, 0.067)	**(0.095, 0.240)**
	sd	7.36	7.532	7.747	7.345	7.544	7.729
		(7.306, 7.415)	(7.486, 7.578)	(7.702, 7.791)	(7.290, 7.400)	(7.498, 7.590)	(7.685, 7.772)
Angle	Concentration	0.076	-0.718	0.452	0.061	-0.724	0.506
		(-0.018, 0.171)	(-0.861, -0.574)	(0.378, 0.527)	(-0.033, 0.157)	(-0.864, -0.583)	(0.435, 0.577)
Max Depth	Intercept	6.175	5.051	3.889	6.188	5.027	3.872
		(6.146, 6.204)	(5.001, 5.102)	(3.817, 3.962)	(6.161, 6.214)	(4.978, 5.076)	(3.801, 3.944)
	**CSI**	0.005	-0.072	**-0.190**	-0.018	-0.006	**-0.097**
		(-0.006, 0.073)	(-0.160, 0.016)	**(-0.295, -0.085)**	(-0.071, 0.033)	(-0.086, 0.073)	**(-0.179, -0.015)**
	sd	4.919	4.842	3.679	4.907	4.848	3.691
		(4.847, 4.990)	(4.791, 4.893)	(3.593, 3.765)	(4.841, 4,927)	(4.797, 4,899)	(3.607, 3.774)
Dive Wiggles	Intercept	5.791	4.494	3.283	5.797	4.488	3.296
		(5.734, 5.847)	(4.441, 4.547)	(3.202, 3.365)	(5.740, 5.853)	(4.435, 4.540)	(3.218, 3.374)
	sd	5.573	4.481	3.318	5.571	4.485	3.336
		(5.501, 5.644)	(4.420, 4.542)	(3.218, 3.417)	(5.500, 5.643)	(4.424, 4.545)	(3.241, 3.431)
	zero mass	-1.292	-2.314	-1.894	-1.283	-2.299	-1.901
		(-1.441, -1.143)	(-2.490, -2.139)	(-2.061, -1.728)	(-1.432, -1.133)	(-2.472, -2.127)	(-2.070, -1.732)
	Intercept	2.139	0.830	1.388	2.132	0.809	1.447

		(2.105, 2.173)	(0.816, 0.844)	(1.348, 1.429)	(2.099, 2.164)	(0.794, 0.824)	(1.406, 1.489)
Distance to Shore	**CSI**	**0.089**	**-0.255**	**0.486**	**0.125**	**-0.225**	**0.311**
		**(0.011, 0.166)**	**(-0.288, -0.221)**	**(0.411, 0.562)**	**(0.060, 0.189)**	**(-0.265, -0.186)**	**(0.250, 0.371)**
	sd	1.411	-0.623	1.027	1.387	-0.550	1.037
		(1.369, 1.454)	(-0.660, -0.586)	(0.985, 1.068)	(1.344, 1.431)	(-0.587, -0.512)	(0.996, 1.078)
